# How are different levels of knowledge about physical activity associated with physical activity behaviour in Australian adults?

**DOI:** 10.1371/journal.pone.0207003

**Published:** 2018-11-28

**Authors:** Sara Veronica Fredriksson, Stephanie J. Alley, Amanda L. Rebar, Melanie Hayman, Corneel Vandelanotte, Stephanie Schoeppe

**Affiliations:** Physical Activity Research Group, Appleton Institute, School of Health, Medical and Applied Sciences, Central Queensland University, Rockhampton, QLD, Australia; University of Maiduguri College of Medical Sciences, NIGERIA

## Abstract

People with knowledge of the benefits of physical activity tend to be more active; however, such knowledge is typically operationalized as a basic understanding that physical activity is ‘good’ for health. Therefore, the aim of this study was to investigate whether there are differences in how detailed a person’s knowledge is about the benefits of physical activity. Participants (*N* = 615) completed an online survey to measure their current physical activity behaviour, as well as their level of knowledge of the benefits and risks of physical (in)activity. The majority of participants (99.6%) strongly agreed that physical activity is good for health, however on average, participants only identified 13.8 out of 22 diseases associated with physical inactivity and over half of participants (55.6%) could not identify how much physical activity is recommended for health benefits. Furthermore, 45% of the participants overestimated, 9% underestimated and 27% did not know the increased risk of disease resulting from inactivity as indicated by the Australian Department of Health. Participants were significantly more active when they correctly identified more diseases associated with physical inactivity and when they overestimated the risks associated with inactivity. Therefore, health promotion initiatives should increase knowledge of the types of diseases associated with inactivity. Low knowledge of physical activity guidelines suggest they should be promoted more, as this knowledge provides guidance on frequency, types and duration of physical activity needed for health.

## Introduction

Regular physical activity reduces the risk of all-cause mortality by 30%, reduces the risk of developing major chronic diseases such as cardiovascular disease by 35%, type 2 diabetes by 42%, colon cancer by 30% and increases life expectancy by up to seven years [1,2]. Regular participation in physical activity also improves general physical health and well-being [3]. The Australian Department of Health recommends that Australian adults aged between 18–64 years engage in at least 150 minutes of moderate intensity physical activity per week, or alternatively, in 75 minutes of vigorous intensity physical activity per week to increase the prospect of experiencing the health benefits [3]. However, the 2014–2015 Australian Health Survey suggested that only 56% of Australian adults are meeting these recommendations [4].

Social cognitive theories such as the theory of planned behaviour [5] and the health belief model [6,7] suggest that knowing about the health implications of physical (in)activity increases intentions to be regularly active. However, people’s general beliefs that physical activity is beneficial for health does not predict much variability in intentions or behaviour [8]. These previous null findings may be the result of the superficial operationalization of ‘knowledge’ that is typically used in studies through one or two items (e.g., “Do you think physical activity is beneficial or harmful?”) [8]. A more nuanced approach into understanding people’s knowledge of physical activity benefits is needed before ruling out that knowledge is important in influencing future activity behaviour.

Chapman and Liberman [9] proposed four different levels of knowledge regarding smoking.

Briefly, level 1 knowledge is ‘having heard that smoking increases health risks’, level 2 knowledge is ‘being aware that specific diseases are caused by smoking’, level 3 knowledge is ‘accurately appreciating the severity and probabilities of developing tobacco-related diseases’, and level 4 knowledge is ‘accepting that the risks inherent in levels 1–3 directly apply to one’s own risk of developing such diseases’ [9]. These four levels of knowledge can also be applied to physical activity, as outlined in Table 1.

**Table 1:** Levels of knowledge regarding benefits and risks of physical (in)activity for health.

**Level 1: Knowing that physical activity is beneficial for health and physical inactivity is harmful to health** At the most elementary level, one can ask whether an individual has ever heard that physical activity is beneficial for health, and in turn, that physical inactivity poses risks to ‘health’ in its widest sense. People might simply be aware that ‘being active is good for health’. **Level 2: Knowing that specific health conditions are related to physical inactivity** Level 2 knowledge involves knowing that a lack of physical activity can lead to particular diseases, such as cardiovascular disease (e.g., heart attacks, strokes), type 2 diabetes, colon and breast cancer. Level 2 knowledge also means knowing about the relationship between levels of activity and certain health risk factors, such as high blood pressure and elevated cholesterol. **Level 3: Knowing exactly how much physical activity is needed for health, and the probabilities of developing physical inactivity related health conditions** Knowledge of how much physical activity (frequency, duration, intensity) is needed to receive health benefits. Furthermore, Level 3 knowledge involves an understanding of the magnitude of the health risks associated with physical inactivity (e.g., 35% increase in CVD). **Level 4: Knowing and accepting that the risks and benefits of physical activity (inherent in levels 1–3) apply to one’s *own* risk of developing such health conditions** Level 4 knowledge involves people agreeing and understanding that their physical (in)activity poses significant risks or benefits to their *own* health.

Morrow et al. [10] examined the prevalence of different levels of knowledge regarding physical activity for health among U.S. adults (*N* = 2002, 62% female, 80% aged between 18–60 years). They found that the majority of people (94%) understand and believe that physical activity is good for one’s overall health (i.e., Level 1 knowledge). However, only 70% of the participants had more specific knowledge regarding the minimum days of physical activity per week required to gain health benefits (i.e., Level 3 knowledge). Furthermore, less than 50% of participants were aware of the U.S. physical activity recommendations of at least 150 minutes a week of moderate-intensity, or 75 minutes a week of vigorous-intensity aerobic physical activity [10] Similar findings were reported by Bennett et al. [11] in U.S. adults (*N* = 2701, 52% female, age *M* = 46 years) and Hui and Morrow [12] in Chinese adults (*N* = 812, 54% female, 90% aged between 18–60 years), where participants were found to have an overall poor understanding of physical activity’s role in disease prevention (i.e., Level 2 knowledge). Further, only 57% of the participants in the Chinese study could correctly identify appropriate physical activity recommendations (i.e., Level 3 knowledge) [12]. To-date, only one study has investigated different levels of physical activity knowledge in Australians [13]. This study examined individual (miss)perceptions in relation to physical activity behaviour and health in 2535 Australian adults. They found that 24% of individuals thought they were meeting the national physical activity guidelines (i.e., Level 3 knowledge), when in fact they were not [13]. Moreover, 46% of the Australian adults who did not meet the physical activity guidelines overestimated the health benefits they attributed to their insufficient level of physical activity participation (i.e., Level 2 knowledge) [13]. More research is needed to develop a better understanding of the levels of physical activity knowledge and the relationship with physical activity behaviour in Australian adults. A greater understanding of what type of knowledge is associated with physical activity behaviour will help inform the development of new or improved effective health promotion interventions.

The aims of this study were to (1) identify different levels of knowledge regarding physical activity for health in Australian adults, and (2) to examine the relationship between different levels of physical activity knowledge and physical activity behaviour. It was hypothesised that the majority of adults will have low levels of knowledge regarding the benefits and risks of physical (in)activity for health beyond Level 1. Further, it is hypothesised that adults with higher levels of knowledge regarding the benefits and risks of physical (in)activity for health will be significantly more active than those with lower levels of knowledge.

## Method

### Participants and procedures

Overall, 615 Australian adults participated in the current study. The participants were recruited online through invitations posted on Facebook and emailed to university students and staff at Central Queensland University, as well as businesses listed in local directories in Queensland, New South Wales, Victoria, and Western Australia. Participants were eligible to partake in the study if they were 18 years or older and currently residing in Australia. Respondents were asked to complete the survey by clicking on a link which directed them to the Survey Monkey website (an online survey development cloud-based platform). Prior to starting the survey, participants were provided with an online information sheet that outlined the study aims, participants’ rights and the research team’s contact details should participants have any questions or concerns regarding the study. Upon reading this information, participants were given the option to commence participation of the study and were asked to provide informed consent to participate in the study through ticking a box if they agreed to the terms and conditions of the study. The participants then proceeded to start the survey which took approximately 10–15 minutes to complete. The survey was divided into three separate sections where the demographic questions, Active Australia Survey, and knowledge of benefits and risks of physical (in)activity were each provided on a separate page. Ethical clearance for the study was obtained from the Central Queensland University Human Ethics Committee in May 2017 (H1704-063). The data collection methods met the terms of service for all websites used as part of this research.

### Measures

#### Demographics

The survey included demographic questions regarding age, gender, height and weight. The participants education was further assessed on three different levels, including low levels of education (i.e., up to year 12; 17.6%), medium levels of education (i.e., trade certificate or diploma; 30.4%), and high levels of education (i.e., bachelor or post-graduate degrees; 52%). Furthermore, the survey included questions assessing level of urbanisation (major city, regional area, rural remote area), as well as current employment status (employed/self-employed, part-time employed/part-time self-employed, and unemployed/home duties/student/retired/pensioner).

### Physical activity behaviour

Adults’ levels of physical activity were assessed using the Active Australia Survey [14]. The Active Australia Survey consists of eight questions designed to measure walking activity, moderate intensity activity (e.g., gentle swimming) and vigorous intensity activity (e.g., cycling). For example, the domain ‘walking’ was assessed through two questions: 1) “In the last week, how many times have you walked continuously, for at least 10 minutes, for recreation, exercise or to get to or from places?” with the response being the amount of times and 2) “What do you estimate was the total time that you spent walking in this way in the last week?”, with the response being hours and minutes. Total physical activity was calculated by summing the time spent walking, completing moderate physical activity and completing vigorous physical activity (the number of vigorous minutes was doubled to account for the additional health benefits) according to manual instructions [14]. The Active Australia Survey has been found to have good construct validity for moderate and vigorous activity (r = 0.86 and 0.95, respectively), as well as excellent reliability (0.71 to 0.86, & Spearman’s Rho of 0.54 to 0.77) [14].

### Levels of knowledge of physical activity for health

Knowledge questions pertaining to the impact of physical activity on health were based on Chapman’s [9,15] questionnaire of levels of smoking knowledge. *Level 1 knowledge* was assessed with the question, ‘In your opinion, is participating in regular physical activity beneficial for people’s health? ‘Would you say that it is…’ (5 response options, from ‘not beneficial at all’ to ‘very beneficial’ with higher scores indicating higher knowledge). *Level 2 knowledge* was assessed through asking the participants to select which health conditions were a result of physical inactivity from a list of options including correct responses such as ‘cardiovascular disease’, ‘diabetes’ and incorrect responses such as ‘skin cancer’. Level 2 knowledge was calculated as a sum of how many health conditions they correctly identified as having benefit from increased activity (from 0 to 21). *Level 3 knowledge* was split into two separate variables (a and b). Level 3a was assessed through the use of a multiple choise question with 5 response options of which only one was correct: ‘To the best of your knowledge, how much physical activity is sufficient to achieve health benefits in adults? Do you think it is…’ with incorrect response options such as ‘30 minutes of vigorous intensity physical activity on at least 2 days a week’ and a correct response option, ‘30 minutes of moderate intensity physical activity on 5 or more days a week’. Level 3a knowledge was coded as a binary ‘correct’ or ‘incorrect.’ Level 3b was assessed using four items asking ‘On a range of 0–100%, by what percentage do you think participating in regular physical activity would reduce a person’s risk of developing cardiovascular disease/colon cancer/depression/Type 2 diabetes?’ The participants chose values from a dropdown box listing the values in ten percent increments (0%, 10%, 20% … 100%). Responses were averaged across the 4 items and participants were coded into 1) do not know, 2) underestimate <27.5%, 3) correct >27.5% & <47.5%, and 4) overestimate >47.5%. These cut offs were based on past research which demonstrate the increased risk of these diseases due to inactivity to range from 30–45%, and allowing a 2.5% room for error either side [1,2]. *Level 4 knowledge* was assessed using four items: ‘In your opinion, would not participating in regular physical activity increase your risk of developing cardiovascular disease/colon cancer/depression/Type 2 diabetes at some point during your lifetime?’ (5 response options, from ‘Yes, a very high risk’ to ‘no increased risk’). Responses were averaged to create a Level 4 knowledge summary score (range of 1–5) with a higher score indicating a higher understanding of personal risk of inactivity.

#### Data analysis

The prediction, that the majority of adults would have low levels of knowledge regarding the benefits and risks of physical (in)activity for health beyond Levels 1 was examined using descriptive statistics for each of the levels of knowledge. A generalized linear model analysis (Tweedie with log link which is applicable to non-negative continuous data that includes a mixture of zeros and positive values) was conducted to test whether adults with higher levels of knowledge regarding the benefits and risks of physical (in)activity for health would be significantly more active than those with lower levels of knowledge. The independent variables were the four different levels of knowledge (level 2, level 3a, level 3b, level 4), and the dependent variable was weekly minutes of physical activity behaviour. Age, gender and education were included in the model as covariates.

## Results

In total, 615 participants, 150 males (24.4%), 463 females (75.3%), and 2 individuals identifying as ‘other’ (0.3%) participated in the online survey conducted between May and July 2017. Table 2 presents participant demographics. The participants were between 18 and 77 years of age (*M* = 43, *SD* = 13), with the majority (67%) of the participants residing in regional areas. Participants were of various educational backgrounds including low levels of education (17.6%), medium levels of education (30.4%), and high levels of education (52%). The majority of participants were employed in full-time paid positions (43.9%), followed by people currently not employed (28.6%), and individuals employed in part-time paid positions (27.5%). Only a small percentage of people (0.4%) were found to not possess Level 1 knowledge. Therefore, Level 1 knowledge was excluded from the general linear model testing associations between levels of physical activity knowledge and physical (in)activity.

**Table 2:** Descriptive statistics (N = 615).

		All^a^ (n = 615)	Male (n = 150)	Female (n = 463)
**Socio-demographic factors**				
Age, M (SD)		43.27 (13.24)	44.62 (14.37)	42.84 (12.87)
Education, n (%)				
	Low	108 (17.6%)	29 (19.3%)	79 (17.1%)
	Medium	187 (30.4%)	46 (30.7%)	139 (30.0%)
	High	320 (52.0%)	75 (50%)	245 (52.9%)
Employment, n (%)				
	Full-time	270 (43.9%)	89 (59.3%)	180 (38.9%)
	Part-time/Casual	169 (27.5%)	25 (16.7%)	144 (31.1%)
	No employment	176 (28.6%)	36 (24.0%)	139 (30.0%)
Urbanisation, n (%)				
	City	182 (29.6%)	53 (35.3%)	129 (27.9%)
	Regional area	410 (66.7%)	95 (63.3%)	313 (67.6%)
	Rural remote area	23 (3.7%)	2 (1.3%)	21 (4.5%)
**Levels of Knowledge**				
Level 1, n (%)				
1, Not beneficial at all		1 (0.2%)	-	1 (0.2%)
2, Not very beneficial		-	-	-
3, Neither beneficial or not		1 (0.2%)	-	1 (0.2%)
4, Beneficial		44 (7.2%)	14 (9.3%)	30 (6.5%)
5, Very beneficial		569 (92.5%)	136 (90.7%)	431 (93.1%)
Level 2, M (SD)		13.8 (3.43)	13.62 (3.18)	13.87 (3.49)
Level 3a, n (%)				
Correct		250 (40.7%)	51 (34%)	198 (42.8%)
Incorrect		342 (55.6%)	87 (58%)	254 (54.9%)
Level 3b, n (%)				
Don’t know		166 (27%)	38 (25.3%)	128 (27.6%)
	Underestimation	54 (8.8%)	18 (12.0%)	36 (7.8%)
	Correct	120 (19.5%)	37 (24.7%)	82 (17.7%)
	Overestimation	275 (44.7%)	57 (38.0%)	217 (46.9%)
Level 4, M (SD)		3.3 (0.89)	2.75 (1.21)	3.35 (0.91)

^*a*^Only two individuals identified their genderas ‘other’. Therefore, the two participants are only included in the calculations for the total group.

As seen in Table 2, levels of knowledge of benefits and risks of physical (in)activity were much lower beyond Level 1. On average, participants were only able to correctly identify 13.8 (*SD* = 3.43) out of 22 diseases associated with physical inactivity (Level 2 knowledge), and with a large portion of adults (55.6%) incorrectly identifying how much physical activity is needed for health (Level 3a knowledge). The majority (80%) of participants failed to correctly identify the probabilities of developing diseases if physical activity guidelines were not met. Specifically, many participants overestimated the health impact of physical activity on diseases (44.7%), whereas a much smaller percentage (8.8%) underestimated. On average, participants scored 3.3 out of 5 for Level 4 knowledge which demonstrates on average the belief of a *moderate* susceptibility to disease in their lifetime if they are inactive.

Table 3 shows the results of the model testing whether knowledge was associated with physical activity behaviour. Knowledge of disease risk of physical (in)activity (Level 2 knowledge) was positively associated with time spent in physical activity (β = 1.03, *p* < .05). A significant association was also found for Level 3b knowledge, such that participants who underestimated the impact of inactivity on disease risk were less active compared to participants who overestimated the risk (β = .67, *p* = < .05). No significant associations were found between Level 3a knowledge (β = -1.06, p = .424), or Level 4 knowledge (β = 1.05, *p* = 294) and physical activity behaviour.

**Table 3:** Associations between different levels of knowledge about physical activity for health and time spent in physical activity.

	β	*b*	B 95% CI	*p*
Levels of knowledge				
**Level 2**	1.03	0.03	0.00, 0.05	.020
**Level 3a**				
Incorrect	1.06	0.05	- 0.08, 0.19	.424
Correct	—	—	—	—
**Level 3b**				
Don’t know	0.99	-0.01	-.018, 0.16	.906
Underestimation	0.67	-0.40	-0.74, -0.11	.008
Correct	0.87	-0.14	-0.32, 0.05	.157
Overestimation	—	—	—	—
**Level 4**	1.05	0.05	-.05, 0.15	.294

## Discussion

The present study aimed to take a more nuanced approach to understanding the degree to which Australian adults understand the benefits and risks of physical (in)activity and whether different levels of knowledge is associated with physical activity behaviour. Previous evidence [8] suggests that general knowledge of the health benefits of physical activity does not predict behaviour well, despite the inclusion of this construct in prominent behaviour change theories. Our evidence suggests this may be because most people only have a vague understanding of the relationship between physical activity and health, and when a more nuanced approach to understanding people’s knowledge is taken, the association to behaviour becomes clearer. As such, the prediction that the majority of adults would have low levels of knowledge regarding the benefits and risks of physical (in)activity beyond Level 1, was supported. This is similar to results presented by Morrow et al. [10], who found that 94% of Americans participating in the study had knowledge that physical activity is good for health (i.e., Level 1 knowledge), but at higher knowledge levels, the percentage of participants with correct knowledge was much lower.

Furthermore, the prediction that adults with higher levels of knowledge would be significantly more active than adults with lower levels of knowledge was confirmed for Level 2 and 3b knowledge. This is similar to the findings of Hui and Morrow [12] who suggested that participants with lower levels of knowledge regarding diseases associated with physical (in)activity were less likely to meet the physical activity guidelines. However, our results indicate that promoting exact figures of increased risk of disease (i.e. 30–40% increased risk of cardiovascular disease, diabetes and some cancers) is unlikely to increase physical activity behaviour, because individuals who overestimated the decrease in health risk from being active were significantly more active than those who underestimated the risk. It may even negatively impact the physical activity behaviour of those who currently overestimate the disease risk reduction of physical activity. Health promotion efforts may be more successful if they promote a greater awareness of the entire range of diseases associated with physical (in)activity (Level 2 knowledge) as this is positively associated with physical activity levels. This finding indicates that health care professionals and campaign directors could benefit from emphasizing more on information regarding specific health conditions related to physical inactivity in health promotion materials.

No significant relationship was found between Level 3a knowledge and physical activity behaviour. Many physical activity promotion messages in Australia focus on the dissemination of the national physical activity guidelines recommended for good health (Level 3a knowledge), however our research demonstrates that this knowledge is not associated with physical activity behaviour. This finding differs from those reported by Plotnikoff et al. [16] who found that Canadian adults’ knowledge of specific physical activity recommendations was higher among those who are more active.

Also, no significant relationship was found between Level 4 knowledge and physical activity behaviour. One potential explanation for this lack of association is that the participants who correctly identified Level 4 knowledge may believe that inactivity will lead to an increased risk of disease for them, however as this risk may not be perceived as urgent they may not see a reason to be active early on. Hall and Fong’s [17] temporal self-regulation theory suggests that people’s willingness to perform a health behaviour is partially determined by how soon they anticipate outcomes (such as health consequences) to occur. Therefore, future research should consider incorporating perceived time to risk outcome into investigations of the link between physical activity behaviour and knowledge of risk/benefits.

### Strengths and limitations

The current study has several strengths, particularly the large sample, as well as a great diversity among participants in terms of employment, age, and urbanisation. Furthermore, to the authors’ knowledge, this is the first study to have examined how different levels of knowledge regarding the benefits and risks of physical (in)activity are associated with physical activity behaviours in an Australian population. The median age of the sample was 43 years, 82% of adults had more than 12 years education and 71% were in paid employment which is relatively respresentative of the general Australian population (38 years, 62% and 88%, respectively) [18,19]. However, our sample included 75% females and 30% living in a city as opposed to 51% females and 67% living in a city among the general Australian population [18,19].

While the study had several strengths, there are a some limitations that warrant attention. One limitation of the current study is the overrepresentation of women, with 75% of the sample consisting of female participants. The findings may therefore not generalise to males. Due to the anonymity and format of the online survey, controlling the percentage of males and females proved difficult. For future research it is suggested that measures are taken to assure a more equal distribution of gender in the sample. This could for example be achieved by conducting a telephone survey similar to the study conducted by Morrow et al. [10]. Another limitation of the current study is the reliance on self-report measures for assessing physical activity levels, as recall and social desirability bias may influence the truthfulness of the answers [20, 21]. While the anonymity of the survey minimises this issue, future research may still consider an objective measure for physical activity, such as accelerometers [22]. Furthermore, the physical activity knowledge questions were newly developed based on the four different levels of knowledge regarding smoking proposed by Chapman and Liberman [9], however, their psychometric properties are unknown. Finally, the cross-sectional design of the current study precludes inference on causal relationships and identification of changes in physical activity knowledge over time as predictor of physical activity behaviour. The latter would be worth exploring in future longitudinal studies.

### Conclusion

The results of the current study provides useful insight into how the different levels of physical activity knowledge are associated with physical activity behaviour. The study supported previous findings of limited knowledge in adult populations beyond a basic understanding that physical activity is good for health, in an Australian population. Findings demonstrated an association between knowledge of diseases associated with inactivity and physical activity and that participants who overestimated the increased risk of disease from inactivity were more active than those who underestimated. It is recommended that physical activity interventions and health promotion initiatives improve knowledge of types of diseases associated with inactivity. However, improved knowledge of the exact increased risk of disease from inactivity, knowledge of the physical activity guidelines and an understanding that inactivity increases one’s own risk of disease is unlikely to have any significant effect on physical activity levels.
